# Proteome data from a host-pathogen interaction study with *Staphylococcus aureus* and human lung epithelial cells

**DOI:** 10.1016/j.dib.2016.03.027

**Published:** 2016-03-19

**Authors:** Kristin Surmann, Marjolaine Simon, Petra Hildebrandt, Henrike Pförtner, Stephan Michalik, Vishnu M. Dhople, Barbara M. Bröker, Frank Schmidt, Uwe Völker

**Affiliations:** a Interfaculty Institute for Genetics and Functional Genomics, University Medicine Greifswald, Friedrich-Ludwig-Jahn-Str. 15a, 17475 Greifswald, Germany; b ZIK-FunGene Junior Research Group Applied Proteomics, University Medicine Greifswald, Friedrich-Ludwig-Jahn-Str. 15a, 17475 Greifswald, Germany; c Institute of Immunology and Transfusion Medicine, University Medicine Greifswald, Sauerbruchstr. DZ7, 17475 Greifswald, Germany

**Keywords:** Epithelial cells, Flow cytometry, Internalization, Host-pathogen interaction, Proteomics, SILAC, *Staphylococcus aureus*

## Abstract

To simultaneously obtain proteome data of host and pathogen from an internalization experiment, human alveolar epithelial A549 cells were infected with *Staphylococcus aureus* HG001 which carried a plasmid (pMV158GFP) encoding a continuously expressed green fluorescent protein (GFP). Samples were taken hourly between 1.5 h and 6.5 h post infection. By fluorescence activated cell sorting GFP-expressing bacteria could be enriched from host cell debris, but also infected host cells could be separated from those which did not carry bacteria after contact (exposed). Additionally, proteome data of A549 cells which were not exposed to *S. aureus* but underwent the same sample processing steps are provided as a control. Time-resolved changes in bacterial protein abundance were quantified in a label-free approach. Proteome adaptations of host cells were monitored by comparative analysis to a stable isotope labeled cell culture (SILAC) standard. Proteins were extracted from the cells, digested proteolytically, measured by nanoLC–MS/MS, and subsequently identified by database search and then quantified. The data presented here are related to a previously published research article describing the interplay of *S. aureus* HG001 and human epithelial cells (Surmann et al., 2015 [1]). They have been deposited to the ProteomeXchange platform with the identifiers PRIDE: http://www.ebi.ac.uk/pride/archive/projects/PXD002384 for the *S. aureus* HG001 proteome dataset and PRIDE: http://www.ebi.ac.uk/pride/archive/projects/PXD002388 for the A549 proteome dataset.

**Specifications Table**

**Table:** 

Subject area	*Biology*
More specific subject area	*Proteomics of host*-*pathogen interactions*
Type of data	*MS data*, *tables*
How data was acquired	*Data*-*dependent acquisition of tandem mass spectra using an LTQ Orbitrap Velos mass spectrometer* (*Thermo Fischer Scientific, Germany*).
Data format	*.raw files*
Experimental factors	*A549 cells were infected with S. aureus HG001 pMV158GFP*. *By fluorescence based cell sorting*, *bacteria were separated from host cell debris or infected host cells were sorted from those which did not carry internalized bacteria at different points in time during infection* (1.5–6.5 h p.i.).
Experimental features	*Bacteria and host cells* (*infected*, *non-infected exposed*, *and non-exposed controls*) *were disrupted*, *proteins were proteolytically digested and analyzed by nanoLC–MS/MS* (*each n*=*3*). *After a database search relative protein abundance changes were quantified over time*
Data source location	*Interfaculty Institute for Genetics and Functional Genomics, University Medicine Greifswald*, *Friedrich-Ludwig-Jahn-Str. 15a*, *17475 Greifswald, Germany*
Data accessibility	*Data is within this article and deposited to the ProteomeXchange Consortium via the PRIDE partner repository with identifiers PRIDE*: http://www.ebi.ac.uk/pride/archive/projects/PXD002384 and PRIDE: http://www.ebi.ac.uk/pride/archive/projects/PXD002388

**Value of the Data**

• We provide data from an internalization approach to allow simultaneous and complementing proteome analysis of host (A549 cells) and pathogen (*S. aureus*) employing a combination of cell sorting and nanoLC–MS/MS.
• The data will be of value to researchers studying proteomics of infection and internalization with *S. aureus* or other pathogenic bacteria.
• For the pathogen the data will also allow assessment of protein synthesis rates and stability after infection due to the pulse-chase approach in which bacteria were labeled with heavy amino acids prior to infection.
• The data provide insights into host response to infection but also to the impact of simple contact with bacteria.

## 1 Data

The data provided in this article are derived from an internalization experiment with *S. aureus* HG001 pMV158GFP and human alveolar epithelial A549 cells. Proteome data of internalized bacteria and infected host cells collected hourly in the first hours post infection are presented.

## 2 Experimental design, material and methods

**Fig. 1:**
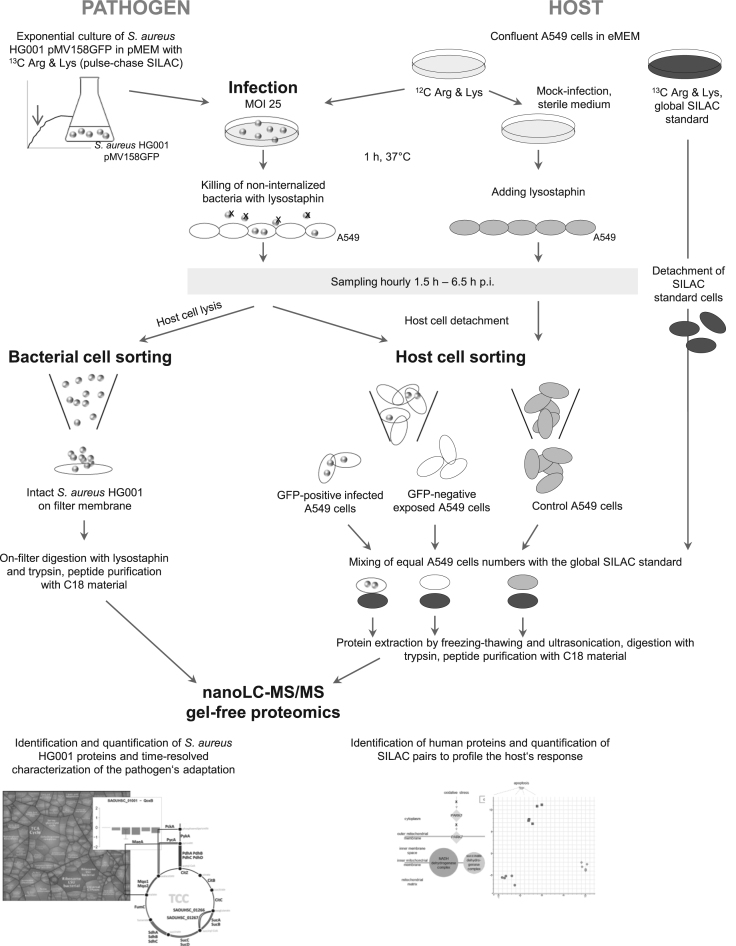
Workflow for proteome data collection of internalized *S. aureus* HG001 pMV158GFP and infected A549 cells. Confluent A549 cells were infected with *S. aureus* HG001 pMV158GFP for one hour and afterwards non-internalized bacteria were killed with lysostaphin. For analyzing bacterial proteins, bacteria were sorted from host cell debris with the help of green fluorescent protein (GFP). To monitor host cell proteins, whole A549 cells were detached from the cell layer and GFP-positive infected A549 cells were separated from GFP-negative exposed A549 cells. Also a group of mock-infected control A549 cells was prepared. Proteins were extracted from bacteria or host cells prior to tryptic digestion and subsequent nanoLC–MS/MS data acquisition. After database search, intensities of bacterial proteins were relatively quantified over time. Quantification of host cell proteins and comparison of the three different groups was accomplished with a global SILAC standard. MOI: multiplicity of infection; pMEM/eMEM: prokaryotic/eukaryotic minimal essential medium; Arg: arginine; Lys: lysine; SILAC: stable isotope labeling of amino acids in cell culture; LC: liquid chromatography; MS: mass spectrometry; GFP: green fluorescent protein.

The workflow used to generate proteome data of internalized *S. aureus* HG001 pMV158GFP and infected A549 cells was published previously [1] and is summarized in Fig. 1.

### 2.1 Cultivation of bacteria

*S. aureus* HG001 [2] carrying plasmid pMV158GFP [3] for continuous production of GFP was employed to generate the data. For host cell proteome experiments, bacteria were cultivated in prokaryotic minimal essential medium [pMEM: 1× MEM without sodium bicarbonate (Invitrogen, Karlsruhe, Germany), supplemented with 1% non-essential amino acids (PAA Laboratories GmbH, Pasching, Austria), 4 mmol/L l-glutamine (PAA), 10 mmol/L HEPES (PAA), and 2 mmol/L of each l-alanine, l-leucine, l-isoleucine, l-valine, l-aspartate, l-glutamate, l-serine, l-threonine, l-cysteine, l-proline, l-histidine, l-phenylalanine, and l-tryptophane (Sigma-Aldrich); pH 7.4; sterile filtered] as described before [4]. To ensure stable growth, bacterial pre-cultures were incubated in serial dilutions at 37 °C and 220 rpm overnight in medium containing 20 µg/mL tetracycline and 0.01% of yeast extract (Becton Dickinson, Heidelberg, Germany). A bacterial culture growing exponentially was inoculated into fresh pMEM medium to OD_600 nm_0.05 and cultivated to OD_600 nm_0.4 before host cell infection.

For experiments aiming to analyse *S. aureus* HG001 proteins, bacteria were grown in SILAC pMEM containing ^13^C arginine and ^13^C lysine (Euriso-Top GmbH, Saarbrücken, Germany) instead of light isotopes, as described previously [4]. After cultivation overnight and in a subsequent culture until OD_600 nm_0.4, bacteria were fully labeled and could be used to infect A549 cells in a pulse-chase approach.

### 2.2 Cultivation of eukaryotic cells

A549 cells, an established model cell line which was isolated from a human lung cancer [5], were cultured at 37 °C in a humid atmosphere with 5% CO_2_ in eukaryotic minimal essential medium [eMEM, 1× MEM without arginine and lysine (Customer formulation, PromoCell GmbH, Heidelberg, Germany) supplemented with 4% fetal calf serum (FCS; Biochrom AG, Berlin, Germany, tested for low arginine and lysine content), 4 mmol/L l-glutamine (PAA), 1% non-essential amino acids (NEAA; PAA), and 30 µg/mL ^12^C arginine and 70 µg/mL ^12^C lysine (Sigma-Aldrich, St. Louis, USA)]. In addition, heavy A549 cells were generated as a standard for relative SILAC quantification by replacing arginine and lysine with their heavy (^13^C) isotopes (Cambridge Isotope Laboratories, Inc., Andover, MA, USA) in equal concentrations in the respective SILAC eMEM cell culture medium. Three days before an infection experiment host cells were seeded in 24-well tissue culture plates and cultured at 37 °C in a humid atmosphere with 5% CO_2_ until to obtain a confluent cell layer.

### 2.3 Internalization assay

The internalization assay was performed as described before [4]. In brief, confluent A549 cells were infected with exponentially growing *S. aureus* HG001 pMV158GFP (OD_600 nm_ 0.4) to a multiplicity of infection (MOI) of 25 and co-cultivated for one hour at 37 °C in a humid atmosphere with 5% CO_2_. For analysis of host proteins, bacteria cultivated in pMEM were used for infection. For analysis of bacterial proteins, heavy SILAC labeled bacteria were employed to infect A549 cells in a pulse-chase manner to allow investigation of *S. aureus* protein synthesis and stability. Afterwards, non-internalized bacteria were killed by replacing the medium with fresh eMEM containing 10 µg/mL of the antibiotic lysostaphin (Ambi Products, LLC, Lawrence, NY, USA), and cells were cultivated for at least further 30 min. Bacteria but also infected and exposed host cells were harvested hourly until 6.5 h after infection. In addition, control A549 cells were mock-infected with sterile infection mix containing pMEM (pH 6.95, comparable to bacterial medium at exponential growth phase) instead of bacterial culture. All other steps of the experiment were performed in the same fashion as with *S. aureus* exposure.

Three different internalization assays were performed with each three independent biological replicates: (i) for investigation of the *S. aureus* adaptation to internalization, (ii) for analyzing the host response to bacterial exposure and infection, and (iii) as negative control for the internalization setup.

### 2.4 Preparation of internalized *S. aureus* HG001 for nanoLC–MS/MS

For profiling of bacterial proteins, host cells were lysed with 0.1% Triton X-100 as described before [4]. GFP producing bacteria were separated from host cell debris with a FACSAria IIIu (Becton Dickinson Biosciences, San Jose, CA, USA) with a 488 nm laser exciting GFP (emitted fluorescence detected at 515–545 nm). Isolated bacteria were sorted onto a low protein binding polyvinylidenfluorid (PVDF) membrane filter (0.22 µm pore size) in a 96-well microtiter plate (Millipore, Schwalbach, Germany) supported by a vacuum pump (450–550 mbar) for constant removal of the fluid from the microtiter plate. Afterwards, the filter was washed with 200 µL FACSFlow-buffer or phosphate buffered saline (PBS, PAA) and the membrane was cut into four pieces with a scalpel. The four filter pieces were collected in a reaction tube and stored at −20 °C or prepared subsequently for protease digestion.

Bacteria were directly digested on filter membranes by adding 12.33 µL of 20 mmol/L ammonium bicarbonate in water. Subsequently, 1 µL lysostaphin (0.05 µg/µL; Ambi Products LLC, Lawrence, NY, USA) was added before the sample was thoroughly mixed and incubated for 30 min at 37 °C. Afterwards, 6.67 µL trypsin (0.1 µg/µL in 20 mM ammonium bicarbonate buffer; Promega, Madison, WI, USA) were added to the sample which was again carefully mixed and incubated for 16–18 h at 37 °C. The trypsin reaction was stopped with trifluoroacetic acid at a final concentration of 0.1%. After incubation for 5 min at 37 °C followed by centrifugation (10 min, 13,000×*g*) the supernatant containing peptides was separated from the filter and transferred to a new vial. Solutions were purified from salts by peptide binding to C18-ZipTip columns (Merck Millipore, Billerica, MA, USA), washing, and eluting in 5 µL 50% acetonitrile (ACN) in 1% acetic acid and 5 µL 80% ACN in 1% acetic acid. Samples were dried in a Speed-vacuum centrifuge (Eppendorf, Hamburg, Germany) and reconstituted in a buffer containing 2% ACN and 0.1% acetic acid in HPLC-grade water (J.T. Baker, Center Valley, PA, USA) prior to nanoLC–MS/MS data acquisition.

### 2.5 Preparation of A549 cells for nanoLC–MS/MS

For experiments conducted to monitor the host cell response, A549 cells were not lysed, but detached from the culture plate and separated from the cell layer by adding trypsin-EDTA (PAA) for 7 min at 37 °C and subsequent inactivation of trypsin with eMEM. Medium was removed after centrifugation (500×*g*, 4 °C, 5 min) and cell pellets were resuspended in PBS. Infected and mock-infected A549 cells cultured in eMEM with ^12^C arginine and ^12^C lysine were subjected to cell sorting.

Heavy-isotope labeled A549 cells grown in SILAC eMEM were likewise detached from the plates and the cell layer but not sorted and were collected as spike-in standard for all groups of sorted A549 cells (infected, exposed, and control).

Infected host cells containing GFP producing bacteria (GFP-positive infected host cells) were separated from cells without bacteria (GFP-negative exposed host cells) by cell sorting with the FACSAria IIu. GFP fluorescence of the internalized bacteria was used to discriminate between infected and non-infected cells with the same lasers mentioned above. Sorted host cells were transferred into 15 mL reaction tubes, centrifuged (500×*g*, 4 °C, 5 min), and counted in reduced volume of PBS in a Neubauer chamber. Also the mock-infected control A549 cells were subjected to cell sorting applying the same settings.

Infected, exposed, and control host cells were spiked with equal numbers of SILAC standard A549 cells and centrifuged (500×*g*, 4 °C, 5 min). Host cell pellets were reconstituted in aqueous 2 mol/L thiourea and 8 mol/L urea buffer and immediately frozen in liquid nitrogen. Protein extraction was accomplished by five freezing (liquid nitrogen)-thawing (shaking at 30 °C) cycles supported by 3×3 s ultrasonication at 50% power (SonoPuls, Bandelin electronic, Berlin, Germany) on ice. The soluble protein content of the supernatant after centrifugation (16,000×*g*, 45 min) was determined with a Bradford assay (Biorad, München, Germany). For each sample 2 µg protein were prepared for nanoLC–MS/MS data acquisition by reduction with 2.5 mmol/L dithiothreitol (DTT; 60 °C, 1 h) and alkylation with 10 mmol/L iodoacetamide (IAA; 37 °C, 30 min) followed by trypsin digestion (trypsin:protein=1:25 at 37 °C, 16–18 h, stopped with 1% acetic acid) and purification of the peptide solutions using µC18 columns (Merck Millipore).

### 2.6 Proteomics data acquisition by nanoLC–MS/MS

Purified peptides were separated on a Proxeon Easy nanoLC (Proxeon Biosystems A/S, Denmark) using an analytical column, Acclaim PepMap 100 [C18, particle size 3 μm, 100 Å (LC-Packings, Dionex, USA)] of 15-cm bed length and a solvent system comprising buffer A [2% acetonitrile in HPLC-grade water (Baker) with 0.1% acetic acid] and buffer B (acetonitrile with 0.1% acetic acid). Peptides were enriched on a pre-column, Biosphere C18 (ID 100 µM, particle size 5 µm, length 20 mm, pore size 120Å, NanoSeparations, Netherlands), eluted with a flow rate of 300 nL/min and a solvent gradient of buffer A and B (2–5% B in 1 min, 5–25% B in 59 min, 25–40% B in 10 min, 40–100% B in 8 min). MS/MS analyses of ionized samples were performed on an LTQ Orbitrap Velos mass spectrometer (Thermo Fischer Scientific, Germany) operated in data-dependent mode to automatically switch between Orbitrap-MS and LTQ-MS/MS acquisition. Survey full scan MS spectra (from *m*/*z* 300 to 1700) were acquired with the Orbitrap in a resolution of *R*=30,000. Sequential isolation of up to 20 most intense ions was allowed, and depending on signal intensity, they were subjected to fragmentation in the linear ion trap by collision-induced dissociation. Target ions were selected with an isolation width of 2 Da for MS/MS and dynamically excluded for 60 s. Further settings included electrospray voltage of 1.6–1.75 kV, no sheath and auxiliary gas flow, ion selection threshold of 2000 counts for MS/MS, an activation *q*-value of 0.25 and activation time of 10 ms for MS/MS with normalized collision energy of 35%. The charge state screening and monoisotopic selection was enabled rejecting states +1, +4, and higher along with unassigned charge states.

### 2.7 Database search

Identification of staphylococcal and human proteins was accomplished with the Rosetta Elucidator software (Rosetta Biosoftware, Ceiba Solution Inc., Boston MA, USA) by searching against a database restricted to *S. aureus ssp. aureus* NCTC 8325 entries from NCBI or a Uniprot/SwissProt database from 2012 restricted to human proteins relying on the SEQUEST algorithm rel. 3.5 (Sorcerer built 4.04, Sage-N Research Inc., Milpitas, CA, USA). The alignment search was performed in a distance of 4 min retention time and 10 ppm instrument mass accuracy. Oxidation of methionine and SILAC labeling of arginine and lysine (+6.02 Da, maximum three SILAC labels per peptide, 20 ppm and 0.5 min retention time tolerance) were set as variable modifications. Carbamidomethylation of cysteine was set as fixed modification only in the human search. Peptides were annotated according to Peptide Teller allowing a predicted error of maximum 0.01.
