# Supplementary material for: Proteome data from a host-pathogen interaction study with Staphylococcus aureus and human lung epithelial cells
Source: Data Brief. 2016 Mar 19;7:1031–7. doi: 10.1016/j.dib.2016.03.027 (PMC5063755; doi:10.1016/j.dib.2016.03.027)
Supplement: Supplementary file 1 — Supplementary material [file mmc1.docx]

**Conflict of interest**

The authors of the article “Proteome data from a host-pathogen interaction study with *Staphylococcus aureus* and human lung epithelial cells” submitted to *Data in Brief* have no financial conflicts of interest for the studies described in this manuscript.
